# Statistical Analysis of Dual-task Gait Characteristics for Cognitive Score Estimation

**DOI:** 10.1038/s41598-019-56485-w

**Published:** 2019-12-27

**Authors:** Taku Matsuura, Kazuhiro Sakashita, Andrey Grushnikov, Fumio Okura, Ikuhisa Mitsugami, Yasushi Yagi

**Affiliations:** 1 Osaka University, Osaka, Japan; 2 Hiroshima City University, Hiroshima, Japan

**Keywords:** Diseases, Diagnosis

## Abstract

Traditional approaches for the screening of cognitive function are often based on paper tests, such as Mini-Mental State Examination (MMSE), that evaluate the degree of cognitive impairment and provide a score of patient’s mental ability. Procedures for conducting paper tests require time investment involving a questioner and not suitable to be carried out frequently. Previous studies showed that dementia impaired patients are not capable of multi-tasking efficiently. Based on this observation an automated system utilizing Kinect device for collecting primarily patient’s gait data who carry out locomotion and calculus tasks individually (i.e., single-tasks) and then simultaneously (i.e., dual-task) was introduced. We installed this system in three elderly facilities and collected 10,833 behavior data from 90 subjects. We conducted analyses of the acquired information extracting 12 features of single- and dual-task performance developed a method for automatic dementia score estimation to investigate determined which characteristics are the most important. In result, a machine learning algorithm using single and dual-task performance classified subjects with an MMSE score of 23 or lower with a recall 0.753 and a specificity 0.799. We found the gait characteristics were important features in the score estimation, and referring to both single and dual-task features was effective.

## Introduction

According to the recent statistics, the number of elderly people suffering from memory loss, impaired judgment, reduced concentration and other symptoms of brain dysfunction caused by dementia is more than 4.6 million in Japan^1^ and more than 46 million globally^2^. Dementia mostly affects older people and the risk of being affected increases with age, yet it also occurs at younger ages primarily due to cerebral vascular disease.

Dementia is a broad category of brain diseases that includes Alzheimer’s disease, vascular dementia, Lewy body dementia, Parkinson’s disease, and others. Many types of those diseases are progressive, meaning that initial symptoms are weak and gradually become more prominent with time^3^. Despite significant efforts in developing new treatments to combat dementia, there is no known infallible cure^4^. The therapies used to treat the patients involve psychological therapies, cognitive and behavioral interventions, and medication to treat behavioral symptoms^5–8^. There is evidence that early treatment of dementia though not able to cure the patient helps to deal with daily life routines and in some cases slows down the progress of the affliction^9^. Thus it is crucial to determine the slight decline in cognitive abilities—mild cognitive impairment (MCI)—that are not noticeable by individuals experiencing them or to other people as early as possible. The primary symptoms of dementia include a deficit of memory, failure to understand concepts and impaired judgment behavior. Other signs of the affliction include the decrease of ability to perform locomotion tasks and carry out cognitive assignments such as solving calculus problems^10–13^.

The conventional methods for detecting dementia rely on physical examinations. The most commonly used screening techniques include blood tests, electrocardiography, and X-ray imaging^14–16^. These procedures require highly specialized equipment and trained personnel and a significant amount of time which increases the overall cost of conducting the tests. An alternative approach for performing screening that can be used instead of physical examinations are paper tests: such as the Mini-Mental State Examination (MMSE)^14^, the Revised Hasegawa’s Dementia Scale (HDS-R)^17^ and The Montreal Cognitive Assessment (MoCA)^16^, which consists of simple questions related to calculation, memory, and comprehension. For example, it is said that an MMSE score of 23 or lower indicates that a patient has cognitive impairment. These paper tests are simple and low cost, however require time investment and involvement of a questioner. Also, since the questions are fixed, it is not suitable for taking the tests frequently to avoid to memorize the questions.

Existing studies indicate that dementia reduces the ability of a person to perform multitasking. Even when subjects suffering from dementia perform two different tasks simultaneously, due to heavier cognitive load on the brain their performance is lower than one for a single task^18–21^. Two tasks that are performed simultaneously is referred to as dual-task. It was shown that due to the fact that the dual-task simplifies the analysis of both cognitive and body locomotion characteristics it is efficient for early detection of dementia^22^. The existing endeavors of developing effective methods for the analysis of dual-task performance for evaluation of cognitive ability utilized an only simple set of characteristics such as walking speed and conducted tests on a low volume datasets.

The focus of the research studies presented in this paper was to collect a substantial database of dual-task data using an automatic system^23^, and develop an algorithm for estimating a cognitive score and analyse to find important characteristics for the estimation. The automatic dual-task system^23^ requests subject to carry out two single tasks—solving various calculus problems, marching on the spot—consequently, then the dual-task composed of the two previous single tasks carried out simultaneously. During the execution, the gait features of the patient and statistics of responses on calculus problems are collected.

We installed the dual-task system in three elderly facilities. That allowed us to collect a substantial amount of data from 90 patients since 2017, and it was used for experiments where we aimed to build an algorithm that would be capable of predicting an MMSE score of an individual test subject. We also carried out a comparison of the degree of importance of the acquired features. We used Random Forest (RF)^24^, Support Vector Machine (SVM)^25^ and Neural Networks (NN)^26^ for estimating MMSE score and classify as two classes of <24 and ≥24 using the estimated MMSE score. We achieved the best score for MMSE estimation of over 1.5 measured as the sum of Recall and Specificity using a method based on NN classifier, and we found gait characteristics during single and dual-tasking were important features in the classification.

## Objective

The goal of this study is to associate MMSE scores, widely-used for the screening of cognitive impairments, with the subject behaviors during single and dual tasks captured using an automatic acquisition system^23^. To this end, we performed an analysis of the acquired data and designed an approach for automatic classification of subjects with lower MMSE scores. We calculated various gait characteristics and cognitive task performance statistics used as input for machine-learning-based classification algorithms.

## Methods

We analyzed the single and dual-task (gait and calculation) behavior captured by the automatic dual-task acquisition system based on previous study^23^. The acquired data was converted to 12 features related to the gait and calculation performance, and fed into machine learning algorithms to classify subjects with lower MMSE scores. We here introduce the acquisition system, feature extraction, and MMSE score estimation algorithms.

### Dual-task acquisition system

#### Hardware configuration

**Figure 1:**
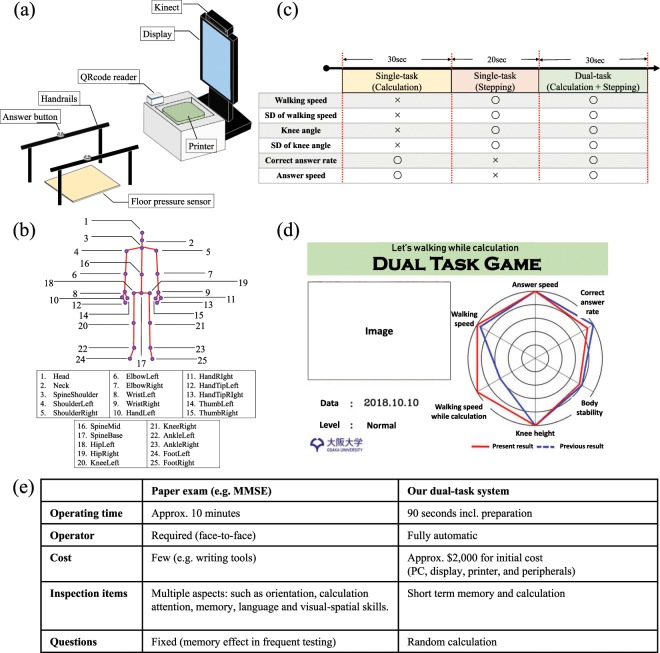
The dual-task acquisition system. (**a**) The hardware configuration of the dual-task acquisition system. The system contains PC, Kinect (version 2), display, QR code reader, floor pressure sensor, handrails with two buttons attached to them. (**b**) Body joints that can be captured with the Kinect device. (**c**) The flow of the implemented dual-task system and the type of the features which are extracted at each game phase. (**d**) Result sheet handed to subjects at the end of the dual-task game. The graph contains six parameters (walking speed while single-task, walking speed while dual-task, the amount of knee height, the amount of body stability, correct answer rate and answer speed). (**e**) A comparison of MMSE and dual-task acquisition system.

For the acquisition of the gait characteristics and statistics of cognitive task solving ability, we used the dual-task system that was introduced in the previous paper^23^. Figure 1 shows a scheme of the dual-task acquisition system. The system consists of Microsoft Kinect v2, PC, display, QR code reader, handrails, floor pressure sensor and buttons that are attached to them (see Fig. 1(a)). Kinect is a motion-sensing input device that can collect the following data: RGB image, the depth map, and body skeleton. We desired to use various features for estimating MMSE score, so we adopted Kinect that can extract body features. Figure 1(b) presents extracted body joints using Kinect. A subject initiates interaction with the system by scanning a unique QR code. The unique ID that is assigned to every subject allows tracking changes in performance that could potentially occur over time in carrying out various single and dual tasks with the passing of time. After the QR code is scanned the subject moves to the designated area where the Kinect device is able to capture the gait data. The subjects in our setup are primarily elderly people, thus to prevent injuries that could occur while carrying out single and dual tasks generated by the system, we installed handrails with buttons attached to them.

#### Tasks

The procedure of data acquisition with the system is separated into three phases: a single cognitive task (30 seconds), a single physical task (20 seconds), and a dual-task (30 seconds). Figure 1(c) illustrates the flow for this system. The subject follows the instructions shown at the display in front of him and first performs two single tasks consecutively - solving calculus problems and walking on the spot. The dual-task consists of gait as the physical task and arithmetic calculation as the cognitive task.

Human gait is a reach source of individual characteristics that was successfully applied to solve various problems such as person re-identification^27,28^, estimating age^29^ and gender^30^. Marching on the spot is the typical form of gait and easy to perform compared with running or walking. As a part of the dual-task paradigm for diagnosis of mental disabilities, the gait features also showed efficiency^31,32^.

Paper tests for evaluating cognitive functions (e.g. MMSE) include cognitive testing in multiple aspects, such as orientation, calculation, attention, memory, language, and spatial skills. Following discussions with psychiatrists, we designed our cognitive task that involves the testing of the calculation ability and short-term memory, both of which are fundamental cognitive abilities. The calculation task is highly related to the *serial-seven* examination included in both MMSE^14^ and MoCA^16^. Because it is challenging to automatically judge the success of serial sevens by automatic systems, we modify the task as a random calculation with two selections. Our calculation questions consist of the addition or subtraction of two numbers. Each question is briefly displayed then replaced by two candidate answers: correct and incorrect ones. The question and answer candidates are not shown simultaneously to load the participant’s short-term memory. The incorrect responses are generated by randomly simulating common mistakes in the calculation: mistakes in carrying and borrowing, unit place, and adding instead of subtracting. Participants hold a button in each hand for selecting the correct answer. Accuracy and response time are recorded, and the participant receives immediate feedback^23^.

After finishing all tasks, six features related the gait and calculation performance were displayed at the screen and also were printed out (see Fig. 1(d)). Subjects could check gait statistics of the current and previous trials. Printing out result sheets would let subjects to be aware that these parameters influence the final score.

#### Comparison with MMSE

We here compare the characteristics of the dual-task acquisition system with MMSE, which is a widely-used cognitive score. The MMSE consists of 11 questions that examine five types of cognitive function: orientation, registration, calculation, recall, and language. The maximum score that represents a healthy adult is 30 points. The score of 23 or lower indicates that a patient has possibly cognitive impairment. Among the advantages of this type of test are simplicity and low cost. As shown in Fig. 1(e), our dual-task acquisition system requires the initial cost for a set of PC, a display, a printer, and peripherals. An important advantage of the dual-task system is quickness (taking approximately 90 seconds) and operator-free (i.e., fully-automatic) acquisition. Together with the characteristics employing random calculation, which does not allow to memorize the questions, the dual-task system is intended to achieve continued and frequent use for finding a drop of cognitive function earlier.

### Data collection

**Figure 2:**
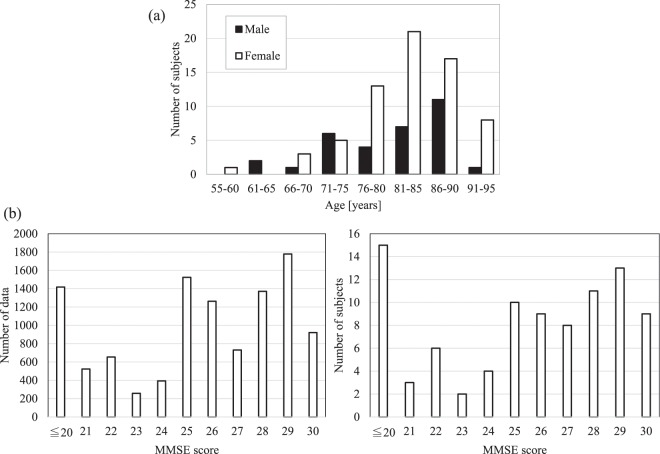
Distribution of acquired dataset. (**a**) Distribution of different age and gender groups in the collected dataset. The total number of males is 32 and females is 68. The average age of a male is 81.5 and of a female is 82.6. (**b**) The distribution of MMSE scores in the collected dataset which includes in total 10,833 data obtained from 90 subjects.

To acquire dual-task gait behaviors from elderly subjects, the dual-task system was installed in three elderly facilities owned by Misasagikai Social Welfare Corporation: Fujidera Assisted Living Facility, Tsudou Elderly Care Center, and Daisen Elderly Care Center since 2017. We recruited subjects from the residents or service-users of three elderly facilities. We limited the subjects who can walk independently, where the decision of the inclusion of each subject was made by the facility staffs. We also confirmed visual functions of subjects are enough to recognize the questions and answers shown on the display. Note we did not select subjects of specific MMSE scores. The system have been operating continuously for over one year, allowing to collect 10,833 data samples from a total of 90 subjects associated with the age and gender of each subject. Figure 2(a) presents the statistics for the subjects from three elderly facilities. Each subject conducted the trials of the system multiple times. The total number of males was 32 and females was 68, while the average age of a male is 81.5 and of a female is 82.6.

The ground truth cognitive score was obtained using MMSE^14^ via face-to-face manner. In the elderly facilities, MMSE score was evaluated for each subject per year, and we collected these score. Figure 2(b) summarizes the MMSE distribution of subjects and captures. The average of MMSE score within 90 subjects was 24.6, and the standard deviation was 5.24; those for 10,833 captures were 25.19 and 4.17, respectively.

This study targets the estimation of MMSE via dual-task behavior analysis; while other paper exams are possible alternatives. In particular, MoCA is reported to perform better classification ability for cognitive impairment, especially for MCI subjects^33^. As the first step of the study, we selected to investigate the relationship between dual-task ability and MMSE, which is the widely-used, gold-standard metric. Since our dual-task employs the calculation task, the performance is thought to be related to the MoCA scores, which include the serial-seven task. An important future direction of this study is to investigate the dual-task ability with other metrics, as well as medical information (e.g. fMRI).

### Feature extraction

During the single- and dual-task data collection, the system captures RGB image, the depth map and skeleton data from the Kinect device, as well as the exact time when the user presses left or right button and step timing on the floor. Since dementia affects the cognitive ability of the patients, their computational ability decreases with the progress of the affliction^10^. Furthermore, other studies provide evidence that gait features, such as walking speed or knee height, are relevant in detecting dementia^12,13^.

Inspired by these observations using obtained data we calculated the following six features for each of single and dual-task: average speed of stepping; the standard deviation of stepping speed; the ratio of correct answers; average time of answering the calculus questions; the average height of knee joint; the standard deviation of height for the knee joint. Figure 1 illustrates obtained features in each phases; in total, we computed 6 × 2 = 12 dimensional features for the analysis.

To calculate the patient’s average walking speed we used the data gathered by the floor pressure sensor. The walking speed was approximated as the time between two consequent steps of left and right foot. This data was utilized to calculate the mean and standard deviation. Next, as a measurement of cognitive ability, we used the ratio of correct answers the patient gave during single and dual tasks, as well as the meantime that was needed to answer the posed question. The answer time was measured from the moment when the two choices were shown at the screen to the instant when the subject pressed one of the buttons.

**Figure 3:**
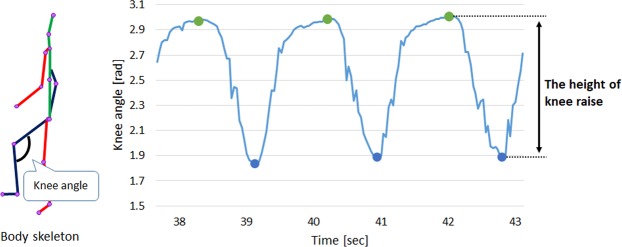
The change of knee angle depending on time. Body skeleton is made by connecting joints together. Knee angle refers to the angle made by three joints: center of the hip, a knee, and an ankle. The green dots are the peak points and the blue dots are the valley points.

As the primary gait characteristic, we exploit the measurement associated with the knee joint. To normalize across different subjects, we compute the knee angle instead of the height of knee raises and performed tracking through time. The height of knee raise was defined as the difference between the lowest and the highest value of the knee angle that are observed at the moments of maximum knee height and at touching the floor as shown in Fig. 3. The angles were measured in radians.

### MMSE score estimation

There are a number of simple yet powerful machine-learning algorithms for performing classification and regression. In this study, we selected to estimate MMSE scores using the following three algorithms: support vector machine (SVM)^25^, random forests (RF)^24^, and neural networks (NN)^26^. The choice of these techniques was motivated by the desire for finding the most prominent features for dementia detection among all other calculated attributes. For that reason, we used three methods SVM, RF, and NN to estimate MMSE score and decide which method is useful for estimating MMSE score, and which features are important.

Even the important aim of this study is to classify the subjects with low MMSE scores, we found that the classification accuracy becomes better when dealing the problem with an MMSE score regression, rather directly solving a two-class classification problem. Therefore, we first estimate an MMSE score from the 12-dimensional feature vectors by solving a regression problem. We then classify the estimated MMSE score as two classes: <24 and ≥24, which is often used for the screening of the possibility of dementia, by thresholding the estimated score. To increase the robustness of the obtained results, the testing phase was performed with leave-one-subject-out cross-validation, i.e., picking up all trials of each subject as testing dataset, and train the machine learning algorithms using the remaining data.

We used the implementations in R programming language: *LiblineaR*, *randomForest*, and *brnn* packages via *caret* interface for effective comparison of the algorithms. The package *LiblineaR* performs a linear SVM; we selected L2-regularized L1-loss functions for score regression. During the training of *randomForest*, which is an RF implementation, we used the default parameters of the function (i.e., creating 500 trees). The *brnn* package implements traditional, two-layers NNs with Bayesian regularization^26^, in which the network parameters are optimized using the Gauss-Newton algorithm. During the training of NNs, we performed a grid search using 10-fold cross-validation within the training examples for selecting the number of neurons from 1 to 3.

The efficiency of the algorithm was evaluated by calculating the discrimination rates for each classification method that were the recall, the specificity, and the sum of recall and specificity.

### Ethics statement

This study was approved by the Research Ethics Committee of the Institute of Scientific and Industrial Research, Osaka University (Osaka, Japan) under the authorization number H29-10. All subjects gave written informed consent. All methods were performed in accordance with the relevant guidelines and regulations.

## Results

### Results of MMSE score regression and classification

Table 1 shows the result of classification into two groups of MMSE scores of <24 and ≥24 via MMSE score estimation using SVM, RF, and NN. The discrimination rate calculated by NN was the highest, and the sum of recall and specificity is over 1.5. SVM notably dropped the accuracy. A possible reason is the SVM implementation, performing linear regression, did not fit the non-linear parameter space. Also, the grid search in NN would effectively work for improving accuracy.

**Table 1:** Binary classification efficiency measurements for different models SVM, RF, and NN.

	Recall	Specificity	Recall + Specificity
SVM	0.602	0.829	1.431
RF	0.688	0.792	1.480
NN	0.753	0.799	1.552

**Figure 4:**
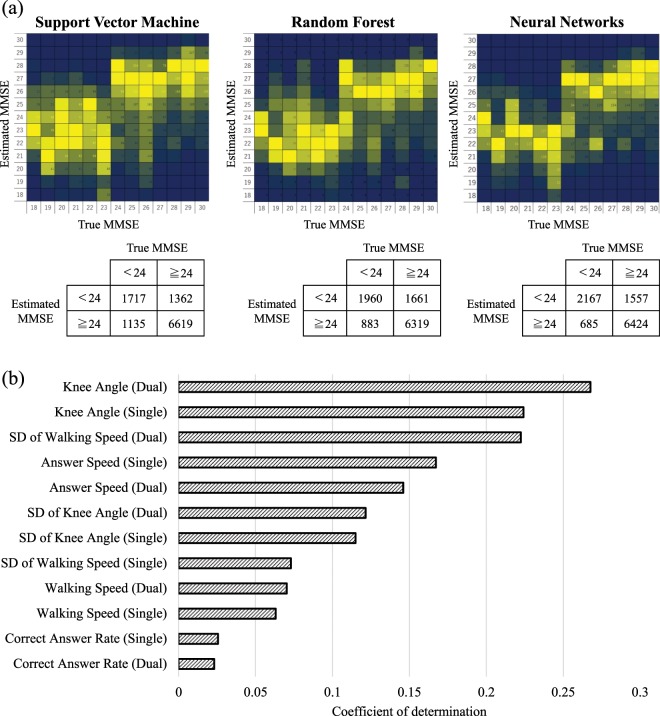
Result of MMSE score estimation. (**a**) Confusion matrix of the MMSE score based on the results estimated by three algorithms. The ratio of the number of subjects of an estimated MMSE score, to the total number of subjects of an actual MMSE score, is illustrated by different colors in the color bar. Warmer colors indicate larger ratios, and colder colors indicate smaller ratios. (**b**) Importance of different characteristics for MMSE score estimation measured with coefficient determination in NNs, which showed the best estimation performance.

For the detailed investigation, Fig. 4(a) visualizes the confusion matrices for the regression and classification of MMSE scores based on the results estimated by NN, which showed the best performance for MMSE score estimation and classification. As the regression result, we only present an MMSE score from 18 to 30 because only a few participants were with the score less than 18. It was found that efficiency of estimating MMSE score changes at MMSE scores 23, 24. It is generally agreed in the medical field that MMSE scores of 23 or 24 are the thresholds that separate dementia and healthy patients. Thus the obtained result confirms that the proposed approach is capable of differentiating between healthy people and people with dementia using the behavioral patterns expressed when carrying out dual-task.

### Importance of features during MMSE score estimation

The importance of each attribute during the training of NN, which were computed using its respective coefficient of determination is shown in Fig. 4(b). These results signify that the standard deviation of walking speed during dual-task and knee angle during single and dual-task were important for the estimation of MMSE scores. Beyond the contribution coefficients computed in the algorithm, we also evaluated the effectiveness of each feature by training NNs using 10-dimensional features except for specific features during single and dual tasks. In Table 2, the classifiers without using walking speed or knee angle dominantly dropped the accuracy. These features were used as key features for the MMSE estimation. Meanwhile, we found the exclusion of some features slightly improves accuracy; the selection of useful features are possible feature direction.

**Table 2:** Classification accuracy without a specific feature.

Recall	Specificity	Recall + Specificity	Recall	Specificity	Recall + Specificity
**w/o walking speed**			**w/o SD of walking speed**		
0.661	0.795	1.455	0.772	0.793	1.566
**w/o knee angle**			**w/o SD of knee angle**		
0.544	0.805	1.349	0.771	0.799	1.569
**w/o answer rate**			**w/o answer speed**		
0.752	0.799	1.551	0.776	0.790	1.566

### Comparison between single-task, dual-task, and combined features

Despite knee angles during a dual task showed higher contribution for MMSE score estimation, the machine-learning algorithms are optimized to use combinations of multiple features. In our case, it is thought effective to combine single and dual-task features to allow to detect the difference such as the performance drop during dual tasks. Table 3 summarizes the comparison of classification ability among single, dual, and combined features when using NNs. From the result, we did not find notable differences in the performance of score estimation between sole use of single task features (a combination of physical and cognitive task features) and the dual-task features. On the other hand, the classification using the combined features of dual- and single-tasks improved the performance, intending the use of both dual- and single-tasks in our paradigm was effective.

**Table 3:** Classification accuracy using single, dual, and combined features.

Recall	Specificity	Recall + Specificity	Recall	Specificity	Recall + Specificity
**Single-task features (physical)**			**Single-task features (cognitive)**		
0.384	0.878	1.262	0.704	0.762	1.467
**Single-task features (physical + cognitive)**			**Dual-task features**		
0.719	0.794	1.513	0.734	0.779	1.514
**Combined features**					
**Recall**		**Specificity**		**Recall + Specificity**	
0.753		0.799		1.552	

## Discussion

From the experimental results, the NN-based machine-learning algorithm performed the best accuracy for MMSE score classification. In addition, knee angle and the standard deviation of walking speed largely contributed to the MMSE score estimation. In this section, we further investigate what was the key factor that makes MMSE score classification possible.

### Investigation of highly-contributed features

**Figure 5:**
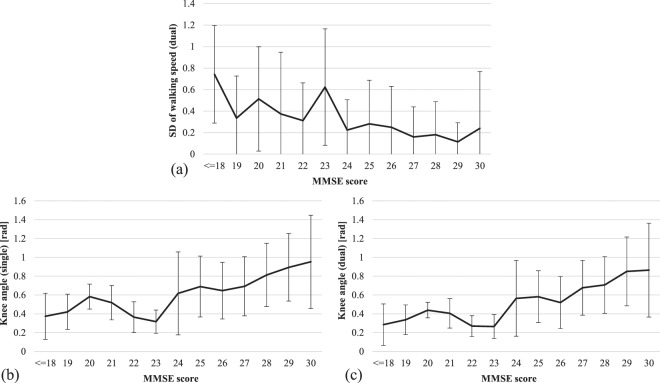
Distribution of three highly-contributed features for MMSE score estimation. (**a**) Standard deviation of walking speed during dual-task. (**b**) Average knee angle during single-task. (**c**) Average knee angle during dual-task.

We checked how the three features with the highest coefficient of determination—standard deviation of walking speed while dual-task and knee angle while single and dual-task—changed in relation to the MMSE score (see Fig. 5). From Fig. 5(a), it is possible to conclude that as the MMSE score increases, the standard deviation of walking speed decreases. This observation can be explained by the fact that healthy people can walk smoothly while executing the dual-task, while people with dementia are not capable of fluid movement when focusing on both tasks simultaneously. The results presented in the Fig. 5(b,c) signify that the MMSE score and knee angle has a close relation: the higher the MMSE score the sharper the knee angle is.

Why knee angles highly related to the MMSE scores? There is a possibility that the machine-learning decisions leveraged the physical ability (e.g. knee angle) to some extent, which may loosely related to their cognitive function. On the other hand, a possible hypothesis is that the subjects who have experienced several trials would notice that he/she should raise his/her knees higher to get a higher score (in the printed result shown in Fig. 1) through those experiences. It would be difficult for those with cognitive impairments to notice it or keep raising knees during a session, particularly when assigning additional cognitive load during the dual-task.

**Figure 6:**
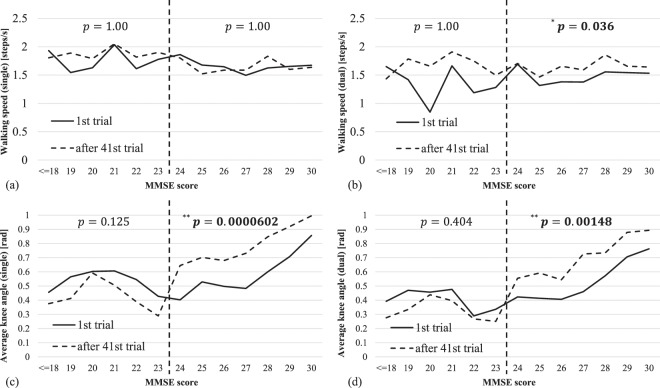
Transition of average walking speed and knee angle: Comparison between the first trial and after 41st trials. (**a**,**b**) Average walking speed (single and dual tasks). (**c**,**d**) Average knee angle (single and dual tasks). The significance value *p* denotes the adjusted *p*-values computed by the Student’s t-test with the Bonfferoni correction (^*^p < 0.05, ^**^p < 0.001).

To investigate if the subjects increased awareness to raising knees after multiple experiences of the dual-task system, we compared the average knee angle during the first trial of each subject to well-experienced trials (the average after 41st trials); where we suppose the subjects did not aware of raising knees at the first trial. Also, we visualized walking speed during a single task, as a baseline of the subject’s physical ability. Figure 6 shows the comparisons. As a simple baseline of walking ability, Fig. 6(a) compare the walking speed during a single task and we did not find a clear trend among different MMSE scores or number of trials. However, as shown in Fig. 6(c,d), the well-experienced subjects with higher MMSE scores raises knee largely compared to the first trial. To confirm the trend, we performed the statistical analysis for each participant group with lower (i.e., <24) and higher (i.e., ≥24) MMSE scores, using multiple Student’s t-tests (two-tailed) with the Bonferroni correction. The participants with higher MMSE scores, indeed, significantly increased the amount of raising knees. We found a similar trend from the knee angles during both single and dual tasks. This result implies the subjects' attention to raise knees, where such attention is somewhat related to cognitive function; and it may increase the classification ability by the machine learning algorithm. From Fig. 6(b), we also see the significant difference of dual-task walking speed for higher-score participants. This may be related to the slight performance drop when excluding the walking speed features, shown in Table 2. At this moment, it is an open question that if the algorithm actually measured the physical ability rather than their cognitive ability (i.e., evaluating if subjects can raise their legs physically), although showing the potential to leverage physical measures during performing designated tasks for the evaluation of cognitive functions.

### Transitions in multiple trials

As discussed above, since all subjects that participated in the dataset acquisition used the dual-task system multiple times, it is possible that the subjects became gradually accustomed to the tasks they were required to perform and the features calculated at the beginning of the trials were different from those obtained at the end. Therefore, we made a comprehensive analysis of the transitions related to the number of trials.

**Figure 7:**
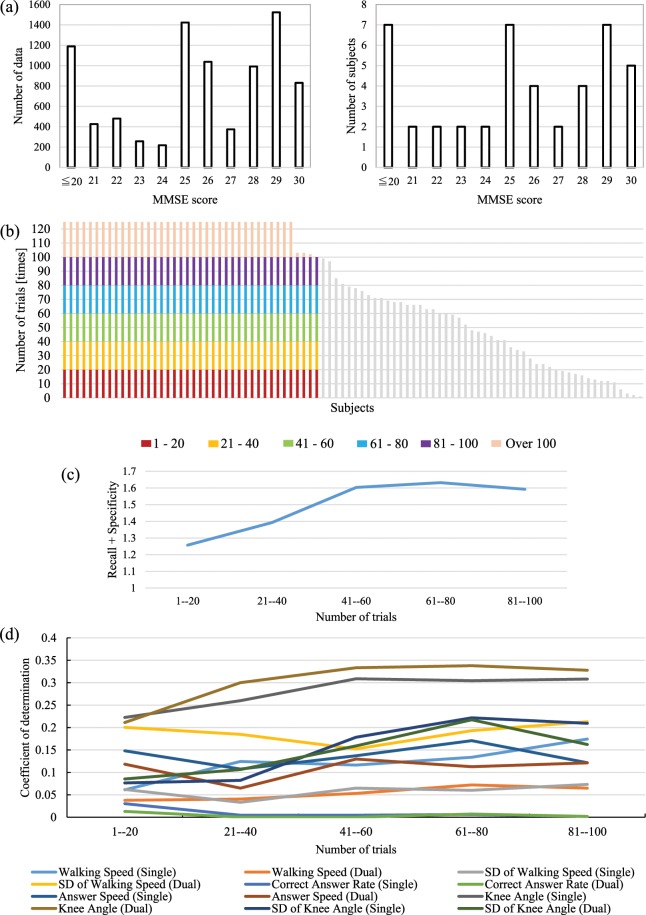
Transition in multiple trials. (**a**) MMSE dataset with the subjects who experienced over 100 times. The dataset includes 8,750 data and 44 subjects. (**b**) From the entire dataset subjects that had done over 100 trials were selected. These data were split into five groups by the number of trials: 1st-20th; 21st-40th; 41st-60th; 61st-80th; 81st-100th. (**c**) The discrimination accuracy increases with the increase in the number of trials subjects conducted previously. (**d**) Degree of the feature importance for MMSE score estimation. The average knee angle (single task and dual-task) importance increases until the number of conducted trials reaches 40 and plateaus after that.

To investigate the relation between the number of trials and the performance score, first, from the entire data pool subjects who conducted more than 100 trials were selected. The MMSE score distribution of the selected dataset is shown in Fig. 7(a). Next the sequences acquired during the trials were divided into five groups depending on the index of the particular trial as shown in Fig. 7(b). The first group contained observation data from the 1-st to the 20th trial; the second group from the 21-st to the 40th trial; the third group from the 41st to the 60th; the fourth group from the 61-st to the 80th; and the final one from the 81st to 100th. Finally to understand how the performance rate changes with the increase of the number of trials for each group the efficiency of classification into two classes (MMSE score <24 and ≥24) was carried out. The classification was done with the NN in the same manner to the other experiments.

Figure 7(c) represent the accuracy of estimation evaluated as the sum of precision and recall depending on the number of trials. It is easy to see that the efficiency of classification gradually increases with the increase in the number of trials, and does not change much after reaching starting from the 40th trial. The investigation of the change of importance of extracted characteristics for MMSE score estimation with the respect of the number of trials (see Fig. 7(d)) showed that the knee angle characteristic during single and dual-task is not only the most important but also that the degree of relevance increases until 40th trial and then plateaus after.

### Improvement of physical function via dual-task training

**Figure 8:**
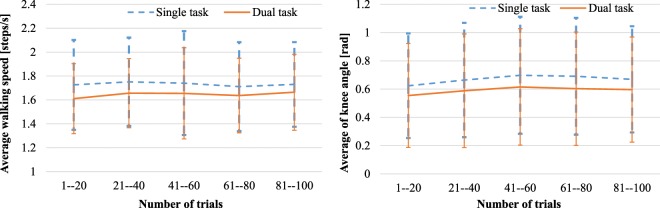
The transition of physical performances during multiple trials of the dual-task system.

Regarding that well-experienced subjects raise their knees higher, it is worth investigating that the consecutive use of dual-task system improves the physical functions. To unveil the improvement of physical ability with the multiple trials of the dual-task system, we visualized the transition of physical measurements for groups of each 20 trials as shown in Fig. 8. Despite the clear difference of the first trial compared to the well-experienced subjects shown in Fig. 6(b), we did not find a clear difference in physical ability depends on the number of trials. Our dual-task system only takes 90 seconds, which is notably shorter than usual rehabilitation training; therefore the system is not directly related to the improvement of their physical ability.

However, we have notified from some individual subjects that the dual-task system would improve their subjective consciousness of the physical ability, since the dual-task increases the general attention for daily physical (i.e., walking) activity. The investigation of the physical aspect (not only cognitive) of the continuous use of the dual-task system is a promising future direction of this study.

## Conclusion

Dementia is a severe affliction that targets elderly people all over the world. Our research is the primary step to develop a fully-automatic framework to evaluate cognitive functions, which is suitable for consecutive uses. The previously designed dual-task system was installed in three elderly facilities for automatic acquisition of rich patient data, including gait characteristics captured with the Microsoft Kinect as well as cognitive measurements in the form of calculation tasks performance statistics. Each elderly person continually conducted trials using the system on a daily basis allowing to collect more than 10,000 trial from 90 subjects.

The main focus of the work presented in this paper was the analysis of the obtained behavior data and designing an approach to detect subjects with low MMSE scores. To this end, we trained machine learning algorithms such as Bayesian Neural Networks to estimate MMSE scores from 12-dimensional features related to physical and cognitive performance during single and dual tasks. We then classify the estimated score into two classes: less than 24 or not, which is often used for screening of possible dementia.

From the result, we confirmed that (1) the combination of single- and dual-task performance was useful for MMSE score classification, and (2) the standard deviation of walking speed during dual-task and knee angle during single and dual-task were important features for MMSE score estimation. Analyzing the change in subjects' performance with the increase of trial times we observed that as the number of trials increases, the discrimination accuracy between high- and low-MMSE subjects also increases. In addition, we found the subjects with higher MMSE scores largely raise knees after a number of trials. From these results, a possible hypothesis is that the subjects who have experienced several trials would notice that he/she should raise his/her knees higher to get higher score displayed by the dual-task system. It would be difficult for those with cognitive impairments to notice it or keep raising knees during a session, particularly when assigning additional cognitive load during the dual-task.

There is room for further investigation about the selection of the type of cognitive assignment that can be used in the dual-task system. At the moment, the system requests patients to solve simple calculus tasks, allowing successfully to recognize people with low MMSE scores based on behavioral and locomotion patterns. We suspect that increasing the task difficulty or introducing a different type of task would allow identifying even less prominent afflictions such as Mild Cognitive Impairment (MCI). Also, an important future direction of this study is to investigate the dual-task ability with other metrics such as MoCA, as well as medical information (e.g. fMRI).

## Data Availability

The datasets used and/or analyzed during the current study are available from the corresponding author on reasonable request.
